# Mean GH profile is more accurate than single fasting GH in the evaluation of acromegaly disease control during somatostatin receptor ligands therapy

**DOI:** 10.1007/s40618-022-01830-6

**Published:** 2022-06-24

**Authors:** C. Bona, N. Prencipe, A. M. Berton, F. Bioletto, M. Parasiliti-Caprino, V. Gasco, E. Ghigo, S. Grottoli

**Affiliations:** Division of Endocrinology, Diabetology and Metabolism, Medical Science Department, University Hospital “Città della Salute e della Scienza di Torino”, Corso Dogliotti 14, 10126 Turin, Italy

**Keywords:** Pituitary, Acromegaly, Somatostatin receptor ligand, Insulin-like growth factor I, Growth hormone

## Abstract

**Purpose:**

This study aims to compare the accuracy of mean GH profile (GHP) < 2.5 ng/ml and single fasting GH (SGH) < 1 ng/ml in the evaluation of disease control in acromegaly patients during somatostatin receptor ligands (SRLs) therapy.

**Methods:**

We retrospectively enrolled 100 acromegaly patients, 68 responder, and 32 partial responder to SRLs. Controlled disease has been defined as IGF-I levels within age-related normal limits, while partial response as pathological IGF-I values despite a reduction ≥ 50%. In all patients, GHP, SGH, IGF-I, and IGFBP-3 were evaluated.

**Results:**

Median GHP levels (1.2 ng/ml, IQR 0.5–2.3 ng/ml) were lower (*p* = 0.001) than SGH (1.9 ng/ml, IQR 1.0–3.6 ng/ml). Accuracy of GHP was 81%, whereas that of SGH was 55%, with a Kappa index of 0.520 and 0.237, respectively. In multivariable analysis GHP (*p* = 0.002) and IGFBP-3 (*p* = 0.004), but not SGH, were independently associated with normal IGF-I levels. At receiver–operator characteristic curve (ROC) analysis GHP cut-off sensitivity and specificity were 94.1% and 50.0%, respectively, while SGH sensitivity and specificity were 35.3% and 93.7%, respectively. Finally, in obese patients the GH cut-off level (both as SGH and GHP) associated to good disease control was significantly different with respect to not obese ones.

**Conclusions:**

GHP associates with IGF-I (and therefore with appropriate control of disease) with higher accuracy than SGH. When GH evaluation is needed, the measurement of mean GHP should be preferred and use of BMI-related cut-offs is suggested.

## Introduction

Acromegaly is a rare disease that is most often caused by a growth hormone (GH) secreting pituitary tumor, associated with several comorbidities (e.g., cardiovascular, metabolic, and osteoarticular diseases), impaired quality of life, as well as increased mortality. Growth hormone and IGF-I are the biochemical parameters used to diagnose acromegaly and to assess disease activity during treatment and last GH and IGF-I levels are fundamental mortality prognostic determinants [1, 2].

Indeed, several studies have related control of acromegaly, defined as a normal IGF-I value [1, 3–5] and/or a GH concentration below a specific cut-off [6–10], to an improved mortality risk and a lower prevalence of several comorbidities. Therefore, the consensus statements are focused mainly on restoration of “safe” GH levels,, which stand for plasma GH level below which the mortality rate approaches that of a normal population, and normalization of IGF-I values [11–13]. The biochemical target for GH concentration has changed over time, while this is not the case for IGF-I, having always been considered those within normal age-matched range.

In 2000, Cortina Criteria [11] established that normal age-matched IGF-I levels and a mean integrated 24-h GH levels of less than 2.5 ng/ml exclude active acromegaly and result in normalization of mortality rates. Moreover some epidemiological studies used a single GH measurement, advancing the idea of a “safe” GH level estimated as being less than 2.5 ng/l [9, 10].

More recent Consensus [14, 15] instead indicated that the best cut-off for GH is random (or fasting) GH < 1.0 ng/ml. These evolving targets are due to mortality studies that demonstrated that lower the GH levels, lower the standardized mortality rate (SMR) [2].

In clinical practice, when both GH and IGF-I are measured, the results do not always yield the same conclusion regarding the response to treatment.

The most important determinant for health, however, is generally considered to be IGF-I, as documented from some studies focusing on discrepancy: in Belgium Registry [16, 17] patients with elevated IGF-I, even when with normal GH, had persistently active disease (deterioration of metabolic profile, radiologic evidence of disease progression, etc.). Moreover, Ronchi et al.[18] demonstrated that, in post-surgery patients with normal IGF-I values, metabolic parameters as well as prognosis were independent from GH levels.

The degree of discrepancy between GH and IGF-I depends at least in part on the cut-off used for the GH measurement: lower the GH cut-off, higher the probability of discrepancy, even though in the study by Machado et al. [19], no relevant reduction in the prevalence of discordance (that had instead been reported in other class of acromegaly patients, such as at diagnosis or after surgery) was observed in patients studied during treatment with octreotide LAR when the cut off level of GH was changed from 2.5 to 1 ng/ml. This was because patients with elevated GH levels and normal IGF-I were still discordant even when reducing GH cut-off.

Another factor to take into account when using single GH measurements (random and/or fasting), is that they can be influenced by a huge biological variability [20] and could possibly not reflect the prevailing daily hormonal output.

As a result of these considerations, aim of our study was to compare the two different “safe” GH cut-off values which were proposed in literature, both as GH profile and single fasting GH, about their reliability in defining treatment control in acromegaly patients under somatostatin receptor ligands (SRLs) therapy.

## Materials and methods

We retrospectively studied 100 acromegalic patients under SRLs followed up in our outpatient clinic. Inclusion criteria were: (1) active acromegaly (diagnosed as elevated IGF-I levels and failure to suppress GH < 1.0 ng/ml at OGTT) due to GH-secreting pituitary adenoma; (2) SRLs treatment for at least 12 months; (3) available GH profile and IGF-I evaluations; and (4) with biochemical evaluations performed in the same laboratory, using the same assays for both GH and IGF-I measurements. Exclusion criteria were: (1) treatment with Pegvisomant, (2) changes in the treatment schedule within last 12 months and (3) severe chronic kidney disease, severe uncontrolled diabetes mellitus, liver disease, malnutrition, as comorbidities that could impact the concordance of IGF-I and GH levels. Patients who did not show response to SRLs were not included, since GH profile was performed only when significant reduction in IGF-I levels was recorded, as in this subset of patient discrepancy between GH and IGF-I status could be challenging for clinicians.

Patients were divided in SRLs responders (SRLs-R; n = 68) and in SRLs partial responders (SRLs-PR, n = 32). Responsiveness was defined as normal IGF-I levels, while partial responsiveness as a reduction in IGF-I values of at least 50% versus pre-treatment ones. All SRLs-R patients were long-term (median 7 years, IQR 6–8 years, range 1–20 years) treated with SRLs (n = 52 with Octreotide LAR, dose range 10 mg/28 days–30 mg/28 days; n = 16 with Lanreotide long-acting dose range 60 mg/70 days–120 mg/28 days; no one of the patients was treated with Pasireotide); the therapy was stable (at the same dosage) for 2–5 years. SRLs-PR patients were under SRLs treatment for 12 months at least at the maximal dose permitted by Italian regulatory rules, at the time of the study.

The study adhered to the principles of the Declaration of Helsinki and was approved by the Ethics Committee of the “A.O.U. Città della Salute e della Scienza”, University Hospital of Turin. All subjects gave their informed consent to participate.

Disease activity control was assessed evaluating IGF-I, IGFBP-3 and mean serum GH from a 5 points profile (every 30’) and SGH levels (considered as the first sample of GHP). Blood samples were collected in the morning (first sample between 08.00 and 09.00 h), after an overnight fasting. All hormonal assays were performed the morning before SRLs administration. Hormonal deficiencies, if present, were treated with specific adequate replacement therapy. No female patients were on oral estrogens (OE) treatment.

All GH and IGF-I were measured by the same assay and in the same laboratory.

Serum GH levels (ng/ml) were measured in duplicate by IRMA (IRMA GH, Beckman Coulter, Czech Republic). The sensitivity of the assay was 0.03 ng/ml. The inter- and intra-assay coefficients of variation (CV) were 9.0–14.0% and 2.4–6.5%, respectively.

Serum IGF-I levels (ng/ml) were measured in duplicate by RIA (SM-C-RIA-CT, DIAsource ImmunoAssays, Belgium) after acid- ethanol extraction to avoid interference by binding proteins. The sensitivity of the method was 0.25 ng/ml. The inter- and intra-assay CV were 6.8–14.9% and 4.5–7.0%, respectively. Serum IGFBP-3 levels (μg/ml) were measured in duplicate by IRMA method (Immunodiagnostic System, UK). The sensitivity of the assay was 0.05 μg/ml. Inter- and intra-assay CV were 6.3–12.4% and 4.85–9.65%, respectively.

Blood glucose was measured on serum by immunometric assays on the chemistry analyzer AU5800 (Beckman Coulter Inc, Brea CA, USA). The sensitivity of the assay was 0.7 mg/dl. The inter- and intra-assay coefficients of variation (CV) were 1.6% and 0.7%, respectively.

Glycosylated hemoglobin (HbA1c) was measured on whole blood using an ion-exchange high-performance liquid chromatography (HPLC) on the D-100 Hemoglobin Testing System (BioRad Laboratories, Redmond, WA, USA). The inter- and intra-assay coefficients of variation (CV) were 1.46% and 0.93%, respectively.

### Statistical analysis

Intergroup comparisons were performed with the Student t-test in case of variables with normal distribution (i.e. IGF-I, BP-3, HbA1c, BMI), while the Mann–Whitney U-test was used in all other cases (i.e. GHP, SGH, age, blood glucose, ring size). The Chi square test and the Fisher’s exact test were used to evaluate the association between categorical variables. Concordance between normal IGF-I and GHP or SGH respectively was determined by Kappa statistic. Univariate analysis were performed in order to define the association between IGF-I and each of all analyzed variables (gender, age, BMI, ring size, neurosurgery, GHP, SGH, IGF-BP3, HbA1c). A logistic regression model was also calculated by considering all variables previously associated to IGF-I levels. Correlation coefficients between BMI and GHP or SGH respectively were calculated using the Spearman rank order R. Assessment of the predictive discrimination of both GHP and SGH values to IGF-I normalization was made using the ROC curve. Then, sensitivity and specificity values for the different GH cutoff values applied in the study were calculated. Furthermore, the best-fitting GH values were computed for both GHP and SGH. For BMI analysis, ANOVA was performed.

Statistical analysis and figures were performed using MedCalc™, version 18.11.3.

## Results

We studied 100 patients with acromegaly in SRLs (72 F; median age 64 years, IQR 53.5–71 years; median IGF-I 209 ng/ml, IQR 158.5–268.5 ng/ml; median IGF-I ULN 0.79, IQR 0.64–1.15; median IGFBP-3 2.6 µg/l, IQR 2.2–3.2 µg/l). Clinical data of patients at diagnosis and at enrollment are reported in Table 1.

**Table 1:** Clinical and hormonal data of acromegaly patients

	All n = 100	SRLs-R n = 68	SRLs-PR n = 32	*p*-value*
Gender (F/M)	72/28	52/16	20/12	0.225
Age at diagnosis (years); mean (IC 95%)	52.2 (49.7–59.7)	51.1 (48.3–54.0)	54.4 (49.3–59.5)	0.230
Adenoma size at diagnosis (mm); median (IQR)	10.5 (9.0–18.0)	11.0 (9.0–16.0)	10.0 (9.0–20.0)	0.914
Macroadenoma at diagnosis (%)	74.0	73.5	75.0	0.930
IGF-I at diagnosis (ng/ml); median (IQR)	634.0 (435.5–893.0)	634.0 (428.0–892.0)	670.0 (472.0–962.0)	0.437
ULN IGF-I at diagnosis; median (IQR)	2.13 (1.38–3.01)	2.01 (1.33–2.82)	2.30 (1.58–4.02)	0.092
GH at diagnosis (ng/ml); median (IQR)	8.1 (4.0–15.0)	8.3 (4.7–15.0)	6.3 (4.0–15.0)	0.340
Pituitary surgery (%)	36.0	43.0	25.0	0.177
Octreotide (%) at enrollment	74	76.5	68.8	0.564
Lanreotide (%) at enrollment	26	23.5	31.2	0.564
Age at enrollment (years); median (IQR)	64.0 (53.5–71.0)	63.5 (53.5–72.0)	64.0 (52.0–67.0)	0.442
IGF-I at enrollment (ng/ml); median (IQR)	209.0 (158.5–268.5)	170.0 (138.0–210.0)	350.5 (258.0–468.5)	**< 0.001**
ULN IGF-I at enrollment; median (IQR)	0.79 (0.64–1.15)	0.70 (0.55–0.79)	1.30 (1.16–1.61)	**< 0.001**
GHP at enrollment (ng/ml); median (IQR)	1.2 (0.5–2.3)	0.9 (0.4–1.5)	2.6 (1.7–4.1)	**< 0.001**
SGH at enrollment (ng/ml); median (IQR)	1.9 (1.0–3.6)	1.5 (0.7–2.7)	3.2 (2.0–4.5)	**< 0.001**
IGF-BP3 at enrollment (µg/l); median (IQR)	2.6 (2.2–3.2)	2.4 (2.0–2.8)	3.2 (2.7–4.1)	**< 0.001**
Blood glucose at enrollment (mg/dl); median (IQR)	93.5 (86.0–111.0)	92.0 (85.0–108.)	95.5 (91.0–115.0)	0.132
HbA1c at enrollment (%); median (IQR)	6.0 (5.7–6.5)	5.9 (5.6–6.4)	6.2 (5.8–6.4)	0.058
BMI at enrollment (kg/m^2^); median (IQR)	26.3 (23.3–28.3)	25.5 (23.3–28.7)	26.8 (24.4–28.3)	0.502

Statistically significant results are given in bold

^*^Comparison between SRLs-R and SRLs-PR

*SRLs-R* responder group, *SRLs-PR* partial responder group, *IGF-I* insulin-like growth factor-I, *GH* growth hormone, *GHP* growth hormone profile, *SGH* single fasting growth hormone, *IGF-BP3* IGF-I binding protein 3, *HbA1c* glycosylated hemoglobin, *BMI* body mass index

We divided subjects in responders to SRLs (SRLs-R, n = 68) and partial responders (SRLs-PR, n = 32) based on IGF-I values. The two groups did not significantly differ for any of the analyzed variables, except than acromegaly disease control (Table 1).

Considering all patients, GHP (1.2 ng/ml, IQR 0.5–2.3 ng/ml) resulted significantly lower (*p* = 0.001) than SGH (1.9 ng/ml, IQR 1.0–3.6 ng/ml).

Accuracy of GHP with a cut-off set at 2.5 ng/ml was 81%, with a Kappa index of 0.520, therefore showing a moderate concordance with IGF-I. Nineteen patients over 100 showed discrepancy, of which 15 as ‘High IGF-I’ pattern and 4 as ‘High GHP’ one.

Accuracy of SGH with a cut-off set at 1 ng/ml was 55%, with a Kappa index of 0.237, therefore showing a poor concordance with IGF-I, lower than the one of GHP. Forty-five patients over 100 showed discrepancy, of which 1 as ‘High IGF-I’ pattern and 44 as ‘High GHP’ one.

In SRLs-R, IGF-I (170.0 ng/ml; IQR 138.0–210.0 ng/ml—ULN 0.70; IQR 0.55–0.79) and IGFBP-3 levels (2.4 µg/l; IQR 2.0–2.8 µg/l) were normal for age. Median GHP levels (0.9 ng/ml; IQR 0.4–1.5 ng/ml) resulted significantly lower (*p* < 0.001) than SGH (1.5 ng/ml; IQR 0.7–2.7 ng/ml). About 94% (64/68) of patients SRLs-R (with normal IGF-I values) had GHP < 2.5 ng/ml and about 35% (24/68) had SGH < 1 ng/ml.

In SRLs-PR, IGF-I (350.5 ng/ml; IQR 258.0–468.5 ng/ml—ULN 1.30; IQR 1.16–1.61) and IGFBP-3 levels (3.2 µg/l, IQR 2.7–4.1 µg/l) were significantly higher (*p* < 0.001) than SRLs-R ones. GHP levels (2.6 ng/ml; IQR 1.7–4.1 ng/ml) resulted similar (*p* = 0.314) to SGH ones (3.2 ng/ml; IQR 2.0–4.5 ng/ml), but significantly higher (*p* < 0.001) than SRLs-R ones (Table 1). Similarly, SGH was significantly higher (*p* < 0.001) than in SRLs-R. About 47% (15/32) of patients SRLs-PR (with pathological IGF-I values) had GHP < 2.5 ng/ml and about 3% (1/32) had SGH < 1 ng/ml.

In univariate analysis (Table 2) we studied the association between GHP, SGH, IGFBP-3, HbA1c, age, body mass index (BMI), ring size (RS), gender and previous neurosurgery (NS) and disease activity control (normal IGF-I). GHP (*p* < 0.0001), SGH (*p* = 0.0004), IGFBP-3 (*p* < 0.001) were negatively associated to normal IGF-I. Blood glucose and HbA1c showed a trend as negative predictors though not statistically significant (*p* = 0.075 and 0.067, respectively). Age, BMI, RS, gender and previous NS did not show association to IGF-I.

**Table 2:** Univariate analysis between normal IGF-I and other variables (GHP, SGH, IGF-BP3, HbA1c, age, BMI, Ring Size, gender and neurosurgery)

Normal IGF-I	Coefficient	Std. error	*p*-value	OR	CI 95% OR
GHP	− 1.070	0.240	**< 0.0001**	0.343	0.214–0.550
SGH	− 0.333	0.107	**0.0004**	0.717	0.580–0.884
IGF-BP3	− 1.500	0.351	**< 0.0001**	0.223	0.112–0.444
Blood glucose	− 0.015	0.009	0.075	0.985	0.968–1.002
HbA1c	− 0.580	0.322	0.067	0.560	0.297–1.053
Age at enrollment	0.016	0.017	0.351	1.016	0.983–1.050
BMI	0.006	0.048	0.902	1.006	0.915–1.105
RS	− 0.056	0.109	0.605	0.945	0.763–1.170
Gender (F)	0.668	0.464	0.152	1.950	0.786–4.839
NS	0.742	0.477	0.110	2.100	0.825–5.347

Statistically significant results are given in bold

*IGF-I* insulin-like growth factor-I, *GH* growth hormone, *GHP* growth hormone profile, *SGH* single fasting growth hormone, *IGF-BP3* IGF-I binding protein 3, *HbA1c* glycosylated hemoglobin, *BMI* body mass index, *RS* ring size, *NS* neurosurgery

Thereafter we performed a multivariable analysis by considering all previously associated variables (GHP, SGH, IGFBP-3, blood glucose and HbA1c). To avoid overfitting, we obtained two different models. In the first one, GHP, SGH, IGFBP-3 and blood glucose were considered (*p* < 0.0001, AUC 0.900, 95% CI 0.823–0.951). GHP (*p* = 0.002) and IGFBP-3 (*p* = 0.004) proved to be negative independent predictors of normal IGF-I. Association of SGH and blood glucose with normal IGF-I did not result significant (Table 3).

**Table 3:** Multivariable regression between normal IGF-I and all variables associated at univariate analysis (GHP, SGH, IGFBP-3, blood glucose and HbA1c), differently combined in fitted models

Normal IGF-I	Coefficient	Std. error	*p*-value	OR	CI 95% OR
GHP	− 1.080	0.348	**0.002**	0.399	0.172–0.672
SGH	0.139	0.181	0.441	1.150	0.806–1.640
IGFBP-3	− 1.18	0.413	**0.004**	0.306	0.136–0.688
Blood glucose	− 0.009	0.013	0.498	0.99	0.967–1.016

Normal IGF-I	Coefficient	Std. error	*p*-value	OR	CI 95% OR
GHP	− 1.061	0.347	**0.002**	0.328	0.168–0.639
SGH	0.127	0.182	0.487	1.135	0.794–1.622
IGFBP-3	− 1.213	0.414	**0.003**	0.297	0.132–0.669
HbA1c	− 0.517	0.434	0.233	0.596	0.255–1.395

Statistically significant results are given in bold

*IGF-I* insulin-like growth factor-I, *GHP* growth hormone profile, *SGH* single fasting growth hormone, *IGF-BP3* IGF-I binding protein 3, *HbA1c* glycosylated hemoglobin

In the second one, GHP, SGH, IGFBP-3 and HbA1c were studied (*p* < 0.0001, AUC 0.900, 95% CI 0.822–0.951). GHP (*p* = 0.002) and IGFBP-3 (*p* = 0.003) proved to be negative independent predictors of normal IGF-I. Association of SGH and HbA1c and normal IGF-I did not result significant (Table 3). Successively, we separately analyzed GHP and SGH to overcome collinearity problem (since SGH was GHP first value). At first, we used a model with GHP, IGFBP-3, blood glucose and HbA1c (*p* < 0.0001, AUC 0.893, 95% CI 0.814–0.946). Both GHP (*p* = 0.0003) and IGFBP-3 (*p* = 0.002) were confirmed to be good negative predictors of normal IGF-I; neither blood glucose nor HbA1c were statistically associated (Table 4).

**Table 4:** Multivariable regression between normal IGF-I and IGFBP-3, blood glucose, HbA1c, GHP (a) or SGH (b)

(a)					
Normal IGF-I	Coefficient	Std. error	*p*-value	OR	CI 95% OR
GHP	− 0.902	0.248	**0.0003**	0.406	0.250–0.659
IGFBP-3	− 1.280	0.414	**0.002**	0.278	0.123–0.626
Blood glucose	0.004	0.016	0.818	1.004	0.972–1.036
HbA1c	− 0.621	0.585	0.289	0.537	0.171–1.693

(b)					
Normal IGF-I	Coefficient	Std. error	*p*-value	OR	CI 95% OR
SGH	− 0.287	0.119	**0.016**	0.750	0.594–0.950
IGFBP-3	− 1.476	0.394	**0.002**	0.228	0.106–0.494
Blood glucose	0.001	0.014	0.970	1.001	0.974–1.028
HbA1c	− 0.653	0.546	0.231	0.520	0.179–1.516

Statistically significant results are given in bold

*IGF-I* insulin-like growth factor-I, *GHP* growth hormone profile, *SGH* single fasting growth hormone, *IGF-BP3* IGF-I binding protein 3, *HbA1c* glycosylated hemoglobin

Secondly, we used SGH, IGFBP-3, blood glucose and HbA1c (*p* < 0.0001, AUC 0.845, 95% CI 0.758–0.910). In this case IGFBP-3 proved to be the best predictor (*p* = 0.0002) while SGH (*p* = 0.0161) had a lower negative predictive power. Neither blood glucose nor HbA1c resulted significantly associated (Table 4).

Finally, we performed ROC curves for normal IGF-I for both SGH and GHP, finding that GHP had a better accuracy than SGH (AUC 0.859, CI 95% 0.775–0.920 vs 0.740, IC 95% 0.643–0.832; difference between areas 0.119, CI 95% 0.061–0.176, *p* = 0.0001).

**Fig. 1:**
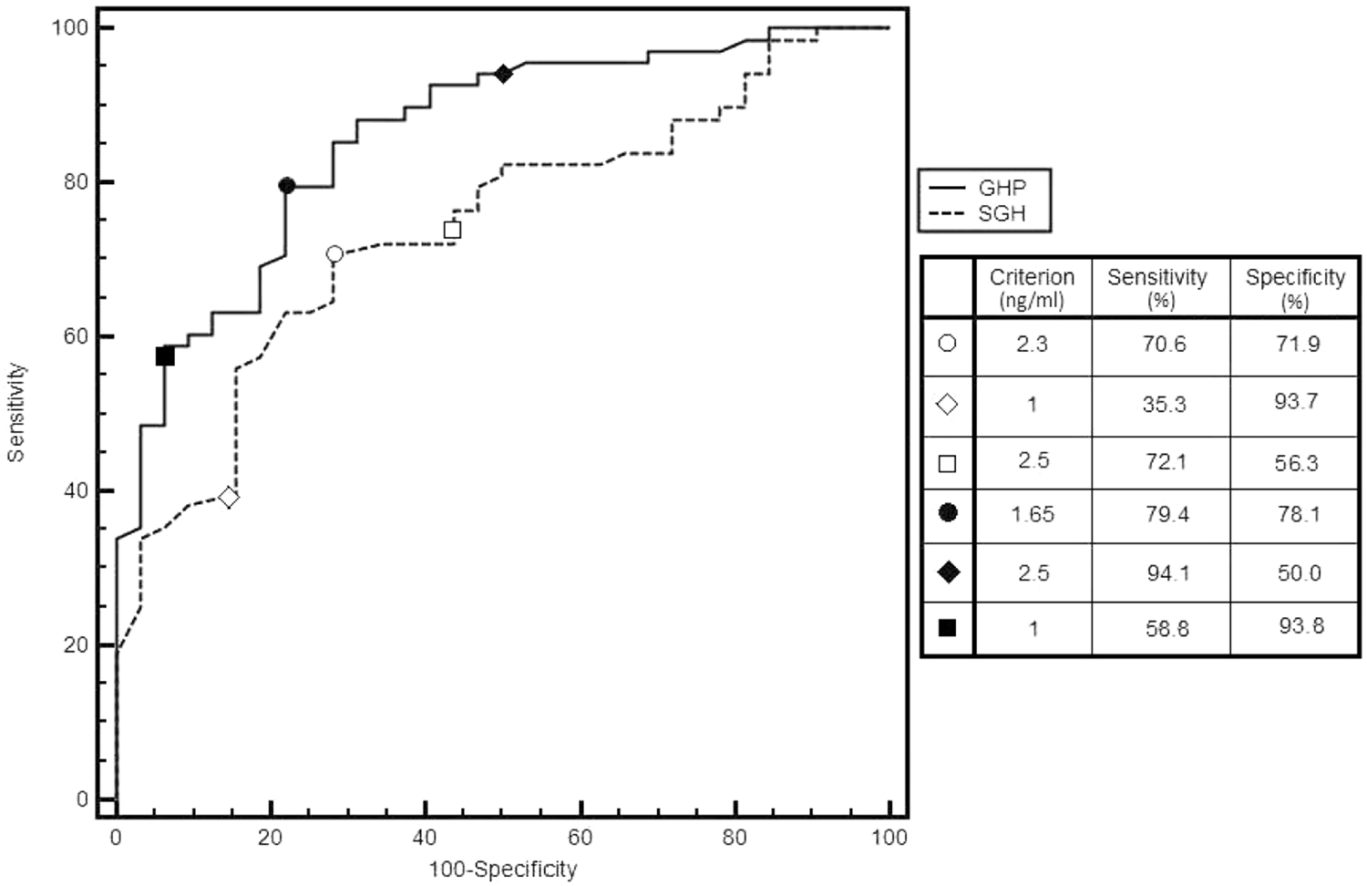
Comparison between GH Profile (GHP) and single fasting GH (SGH) ROC curves

The best diagnostic accuracy of SGH was at 2.3 ng/ml, with a sensitivity of 70.6% and specificity of 71.9%, a positive likelihood ratio (LR) of 2.51 and a negative LR of 0.41 (Fig. 1).

Setting the cut-off for SGH at 1 ng/ml as described in literature, statistical performance was sensitivity of 35.3% and specificity of 93.7%, a positive LR 5.65 and a negative LR 0.69 (Fig. 1).

As far as GHP, the best cut-off was 1.65 ng/ml, with a sensitivity of 79.4% and specificity of 78.1%, a positive LR of 3.63 and a negative LR of 0.26 (Fig. 1). Setting cut-off for GHP at 2.5 ng/ml as described in literature, statistical performance was sensitivity 94.1% of and specificity of 50.0%, positive likelihood ratio (LR) of 1.88 and a negative LR of 0.12 (Fig. 1).

Finally, to test whether the difference in the accuracy between GHP and SGH was effectively related to the sampling protocol (and not only due to the chosen cut-off), we also evaluated accuracy of SGH and GHP setting converse cut-off, which means 2.5 ng/ml for SGH and 1 ng/ml for GHP.

ROC for SGH set at cut-off of 2.5 ng/ml showed a sensitivity of 72.1% and specificity of 56.3%, with a positive LR 1.65 and a negative LR 0.5, with a worst statistic performance than GHP at the same cut-off (sensitivity 94.1% of and specificity of 50.0%, positive LR 1.88 and negative LR 0.12) (Fig. 1).

ROC for GHP set at cut-off of 1 ng/ml showed a sensitivity of 58.8% and specificity of 93.8%, with a positive LR of 9.41 and a negative of LR 0.44, with a better accuracy than SGH for the same cut-off (sensitivity of 35.3% and specificity of 93.7%, positive LR 5.65 and negative LR 0.69) (Fig. 1).

In order to further improve test accuracy, we tried to find confounding variables according to which the diagnostic cut-off should have been adjusted.

Both SGH (Spearman’s coefficient = − 0.220, *p* = 0.029) and GHP (Spearman’s coefficient = − 0.275, *p* = 0.006) showed significant negative correlation to BMI.

**Fig. 2:**
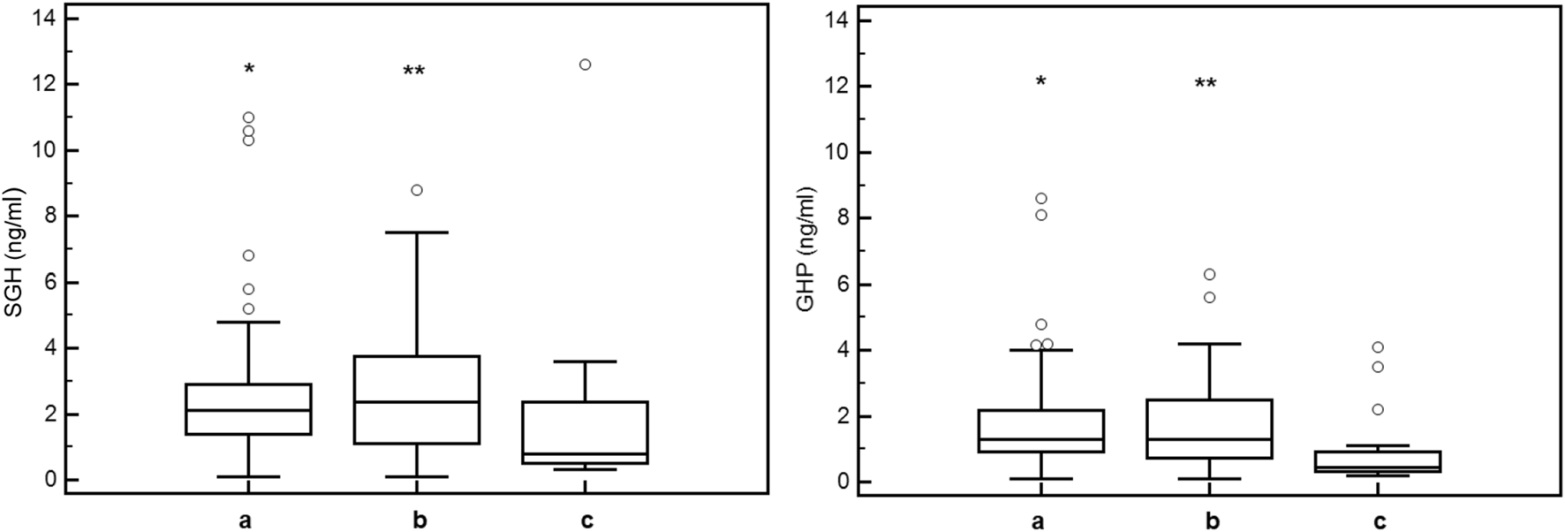
Single fasting GH (SGH) and GH profile (GHP) ranks according to BMI. *p < 0.05 Comparison between Group a and Group c. **p < 0.05 Comparison between Group b and Group c

**Fig. 3:**
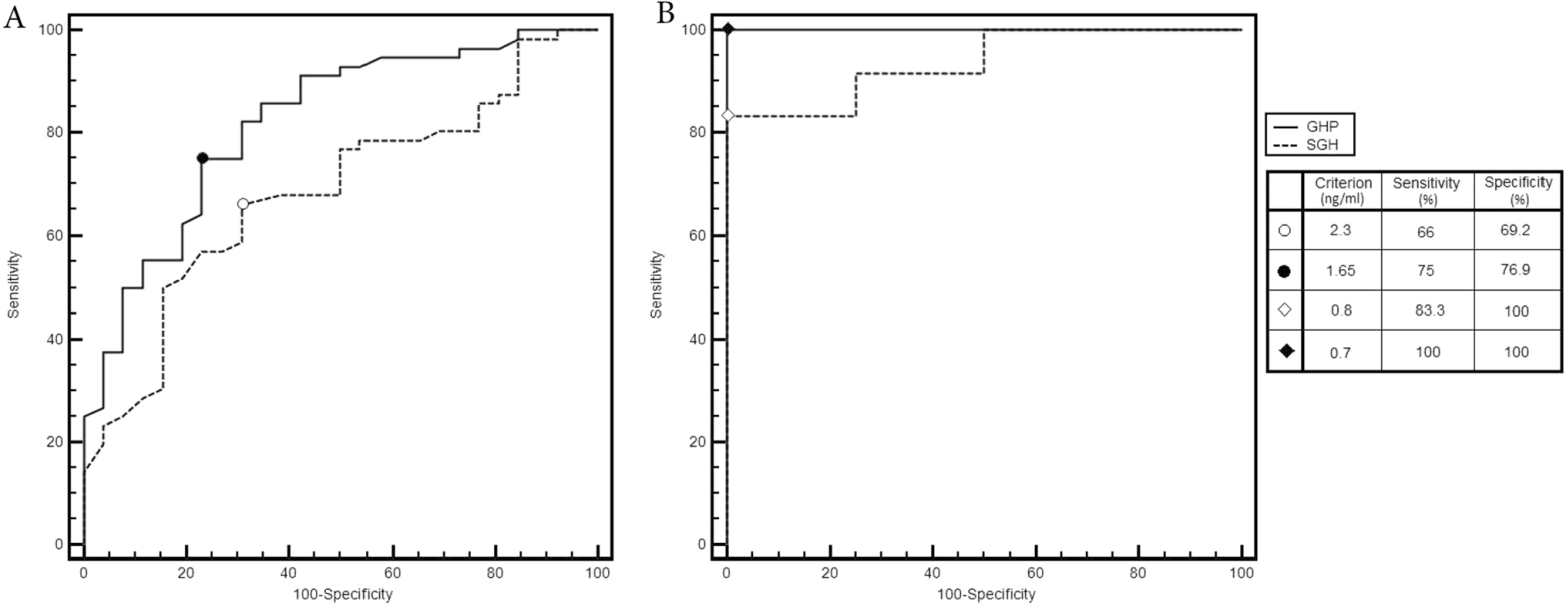
Comparison between GH Profile (GHP) and single fasting GH (SGH) ROC curves, according to BMI: **A** (BMI < 30 kg/m^2^); **B** (BMI ≥ 30 kg/m^2^)

Therefore, as far as SGH, we divided our population in ranks according to their BMI (≤ 25 kg/m^2^, n = 43; 25–30 kg/m^2^, n = 39; ≥ 30 kg/m^2^, n = 18) and we found significant difference among them (*p* = 0.045). At post-hoc analysis, difference was confirmed between normal weight and obese (*p* < 0.05) and between overweight and obese (*p* < 0.05) (Fig. 2). According to this finding, we performed two different ROC curves for SGH in obese patients and not obese patients. In obese patient, the cut-off for SGH with the best diagnostic accuracy was 0.8 ng/ml, (AUC 0.937, sensitivity 83.3% and specificity 100%, positive LR 2.00 and negative LR 0.17), while in not obese patients the SGH best performance is confirmed at a cut-off value of 2.3 ng/ml, (AUC 0.684, sensitivity 66.0% and specificity 69.2%, positive LR 2.15 and negative LR 0.49) (Fig. 3).

**Fig. 4:**
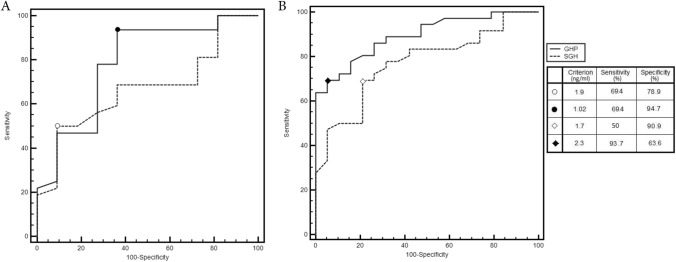
Comparison between GH Profile (GHP) and single fasting GH (SGH) ROC curves, according to BMI: **A** (BMI < 25 kg/m^2^); **B** (BMI ≥ 25 kg/m^2^)

To test whether any grade of weight alteration could exert an action over GH secretion, we repeated ROC curves considering obese and overweight patients together in a unique group. We found that in this subset of studied population the cut-off for SGH which had the best diagnostic accuracy was 1.9 ng/ml (AUC 0.772, sensitivity 69.4% and specificity 78.9%, positive LR 3.3 and negative LR 0.39), while in normal weight patients SGH has the best performance at a cut-off value of 1.7 ng/ml (AUC 0.670, sensitivity 50.0% and specificity 90.9%, positive LR 5.5 and negative LR 0.55) (Fig. 4).

Subsequently we compared how GHP distributed in different groups according to their BMI (≤ 25 kg/m^2^, n = 43; 25–30 kg/m^2^, n = 39; ≥ 30 kg/m2, n = 18) and we found significant difference (*p* = 0.024). At post-hoc analysis, difference was confirmed between normal weight and obese (*p* < 0.05) and between overweight and obese (*p* < 0.05) (Fig. 2). According to this finding, we performed two different ROC curves for GHP in obese patients and in not obese patients. In obese patient the cut-off for GHP, which had the best diagnostic accuracy, was 0.7 ng/ml (AUC 1.000, sensitivity 100% and specificity 100%, positive LR 1.20 and negative LR 0.07), while in not obese patients GHP has the best performance at a cut-off value of 1.65 ng/ml (AUC 0.819, sensitivity 75% and specificity 76.9%, positive LR 3.25 and negative LR 0.32) (Fig. 3).

Repeating ROC curves considering obese and overweight patients together in a unique group, we found that the cut-off for GHP which had the best diagnostic accuracy was 1.02 ng/ml (AUC 0.893, sensitivity 69.4% and specificity 94.7%, positive LR 13.19 and negative LR-032), while in normal weight patients GHP has the best performance at a cut-off value of 2.3 ng/ml (AUC 0.786, sensitivity 93.7% and specificity 63.6%, positive LR 2.58 and negative LR 0.10) (Fig. 4).

Comparing ROC curves for normal IGF-I for both GHP and SGH in not obese patients, GHP still had a better accuracy than SGH (difference between areas 0.135, CI 95% 0.066–0.204, *p* = 0.0001). Considering obese patients only, comparison of ROC curves for normal IGF-I did not show significant difference between GHP and SGH, instead (difference between areas 0.063, CI 95% 0.055–0.180, *p* = 0.298).

Dividing the population into two groups considering overweight and obese patients together, in comparison with normal weight patients, we found that GHP still had a better accuracy than SGH in both groups (normal weight group: difference between areas 0.115, CI 95% 0.002–0.229, *p* = 0.004 and obese/overweight patients: difference between areas 0.121, CI 95% 0.04–0.203, *p* = 0.004).

## Discussion

Our study demonstrates that GH profile has a better accuracy in defining appropriate acromegaly disease control under SRLs treatment than single fasting GH determination, which is less representative. Disease activity can be evaluated in many ways, GH and IGF-I concentrations being the main biochemical markers used to assess the response to treatment. Their levels have been associated with prognosis [21]: in particular lowering GH to “safe” levels and normalizing IGF-I in patients with acromegaly results in mortality rates similar to those expected in the general population, as recommended by guidelines [2, 12, 13]. Although the biochemical target for GH concentration has changed over time, this is not the case for IGF-I that, in everyday clinical practice, is considered the most feasible parameter. By definition, both in the past and nowadays, IGF-I levels are considered normal when within age-adjusted limits. GH cut-off values, instead, have been modified over time for two main reasons: the assays used to assess GH changed over the years (currently ultra-sensitive assays are used) and the accumulated evidence in literature, that had progressively lowered GH value to target for a normalized standardized mortality rate (SMR). According to the earlier criteria [9], biochemical goals to normalize mortality were GH < 2.5 ng/ml or GH nadir during glucose load < 1.0 ng/ml and normal IGF-I levels. Using these limits, Dekkers et al.[5] showed in a metanalysis that the SMR were still increased at 1.09, but the included studies were mainly conducted in patients with acromegaly treated with trans-sphenoidal surgery, while with current effective medical treatments, the SMR outcome could be different. Holdaway et al. [2] demonstrated, in a New Zealand cohort of patients with acromegaly, that a single GH < 1.0 ng/ml (analyzed by ultra- sensitive assays) was associated with normalization of mortality. More recent consensus statements [14, 15] introduced new limits (SGH < 1.0 ng/ml) according to the evidence that lowering GH cut- off decreases mortality rate.

In a systematic review, Bolfi e et al. [22] confirmed that mortality in acromegaly is normalized with biochemical control (generally defined in included studies as normal IGF-I and random GH < 2.5 ng/mL and in one study as IGF-1 levels below 1.2 times the upper limit of normal). Moreover they performed a subgroup analysis according to the treatment modality and found that when SRLs was available as adjuvant therapy, mortality in acromegaly was not different from general population; on the other hand it was significantly higher when only surgery and radiotherapy were available.

In our experience, patients that before 2010 were considered responsive to SRLs (because they showed mean GH* p* < 2.5 ng/ml and normal IGF-I levels), after 2010 frequently had to be considered not well controlled because they showed SGH > 1 ng/ml. This could be due to different reasons.

In acromegaly, GH secretion showed a highly pulsatile nature: therefore, a single random GH sample could possibly not reflect the prevailing daily hormonal output. However, a very detailed paper comparing validity of the intensiveness of GH measurements confirmed good correlations between a single GH measurement and both a mean GH calculated from five samples at predetermined times and a mean GH concentration from 24-h profiles [23]. Anyway, the authors conclude that even though this could be true at estimating a total magnitude of GH hypersecretion in epidemiological studies, random GH is not very reliable in each individual patient.

Moreover, using single GH measurement, it can be influenced by a huge biological variability [20]. It is known that chronic inflammatory disorders, anorexia nervosa, poorly controlled diabetes mellitus, renal failure, hypothyroidism, malnutrition as well as OE treatment [21, 24] can emphasize GH/IGF-I discrepancy. This evidence was confirmed in Belgium acromegaly register [16, 17] in which most of patients who showed GH/IGF-I discrepancy were young female on OE treatment. In our population none of confounding factor was present, in fact no woman was under OE, all diabetic patients had a good glucose metabolism control (median HbA1c 6.0%, IQR 5.7–6.5%) and no renal or liver failure or malnutrition cases were described. The meta-analysis by Kanakis et al. [24] showed, analyzing the data of a large series of treated patients, a discrepancy in GH/IGF-I of 25.7%; the majority concerned the so called “high IGF-I type” (15.3%), whereas the opposite type (“high GH” discrepancy) was observed in 11.1%. The authors underlined that the employment of sharp GH cut-offs resulted in significantly higher discordance rates.

Focusing on patient under medical treatment with SRLs, discordant rates are higher in such a subset of acromegaly population, the predominant pattern of discordance being with high GH and normal IGF-I levels [19, 25, 26]. In this setting, use of the OGTT exhibits an even higher degree of discordance and provides no advantage for assessment of medical therapy outcomes, compared with measuring basal GH levels [25].

Consistently with these results, also Machado et al. [19] described a prevalence of discrepancy due to elevated GH levels and normal IGF-I in patients studied during treatment with octreotide LAR and did not found relevant reduction in the prevalence of discordance when the cut off level of GH was changed from 2.5 to 1 ng/ml, as was instead reported in other class of acromegaly patients (such as at diagnosis or after surgery), since still discordant even when reducing GH cut-off.

A more recent study by Campana et al. suggested using the mean of 3 GH measurements collected during consecutive routine patients’ evaluations, as it mitigates the impact of GH cutoffs on discordance with IGF-I [27].

In our study, SGH < 1 ng/ml showed an accuracy of 55%, GH* p* < 2.5 ng/ml of 81%.

However, as much as 53% of patients with safe GHP levels had pathological IGF-I: in this subset of studied population, mean IGF-I levels showed a trend to be lower (*p* = 0.07) than those of subjects in which concordance between pathological IGF-I and elevated GHP was found. This could mean, probably a lower disease activity. As previously mentioned, discrepancy between GH and IGF-I depends in part on the cut-off used for GH measurement [28–30] and even on availability of highly sensitive and specific new GH assays. Comparison of GH levels measured in traditional versus modern techniques has shown, as expected, that GH levels analyzed by more recent ones are significantly lower than those detected by polyclonal radioimmunoassays (RIA). In addition, the inability to convert between older and newer values is a problem because important epidemiological data, which have been traditionally used as a guide for therapeutic decisions, were derived largely from GH measurements made by polyclonal RIA. The heterogeneity of modern assays, characterized by antibody specificity and different reference preparations [30] is a further complication. The mentioned limitation about variability of SGH can be partially overcome with the use of serial GH samples in the day. Anyway, even the most stringent sampling procedure does not always differentiate between health and disease. In fact, it has been demonstrated that, especially in patients with milder disease activity, mean 24-h GH levels can overlap between active acromegaly subjects and healthy controls [31] suggesting that the quality of secretion is the more important drive for IGF-I synthesis.

Faje et al. indeed demonstrated that, if it is confirmed that plasma IGF-I concentrations correlates with mean 24-h GH concentrations, this relationship is dependent exclusively on the interpulse nadir GH levels and not on GH pulses [32].

Since 24 h GH profile is very time and cost-consuming, various protocols for the collection of GH curves (with no stimulus or suppression) were used by different groups to evaluate GH secretion. Currently there is no consensus for sample collection and different centers have distinct protocol.

Taboada et al. demonstrated a strong correlation both between basal and mean GH, obtained as the arithmetic mean of 5 samples collected with 30 min intervals level in patients receiving SSAs and concluded that there was no benefit to perform GH curves, at least with that protocol [33].

In our study, considering IGF-I value as gold standard, GHP (mean serum GH from a 5-points profile taken every 30’) < 2.5 ng/ml acquired an important role in the evaluation of disease control because of its sensitivity (94.1%). In reverse, SGH < 1 ng/ml demonstrated a high specificity (93.7%) but a low sensitivity (35.3%) and, therefore, it seems not to be that useful as it could no offer further information to clinician in addition to IGF-I. In our population, ROC curves showed the best diagnostic accuracy for GH* p* < 1.65 ng/ml, while the most appropriate cut-off for SGH resulted to be 2.3 ng/ml.

Previous studies found quite similar cut-offs: Cozzi et al. found out that normal IGF-I was predicted by the GH value of 1.8 µg/l in males and 2.4 µg/l in females [26]; according to Campana et al., instead, the best-fitting single fasting GH cut-off was1.63 µg/l [27].

As mentioned before, it could be argued that the better accuracy of GHP in defining disease control could be related to the chosen cut-off, lower for the SGH than for GHP, since the cut-off highly impacts the concordance between GH and IGF-I. However, setting a cut-off for SGH at 2.5 ng/ml showed a worst diagnostic performance than GHP; conversely, GHP cut-off set to 1 ng/ml still had a better accuracy than SGH. Therefore, the difference in the accuracy between GHP and SGH seems to be effectively related to the sampling protocol and not only to the chosen cut-off.

So far as for the comparison between GHP and SGH; however, it is known that other factors, such as BMI, could influence GH secretion, through an inhibitory effect, both in acromegaly and normal patients [34]. Obese individuals show significantly lower GH concentrations at baseline and during many stimulation tests, but also exhibit more pronounced suppression of GH secretion during OGTT in healthy subjects [20, 34]

Vierhapper et al. demonstrated that serum GH measurements obtained during an OGTT in acromegalic patients both before and after transsphenoidal surgery must be interpreted individually, by comparison to control values, taking into account both age and BMI [35].

Indeed, our data confirmed that it could be useful to identify BMI-related cut-off for GHP and SGH also in acromegalic patients under SRLs, to improve their diagnostic accuracy, especially in obese population. Moreover, in such a subset of patients, the difference in accuracy between GHP and SGH seems to be lost.

Our study presents some limitations. First of all, we assumed disease control to be adequately express only through IGF-I levels. Clinical parameters, such as the onset of acromegaly comorbidities or perceived quality of life, could help in better defining disease activity.

Secondly, SGH levels were derived from GHP, being the first sample of the profile.

Thirdly, the used protocol of GH sampling did not allow us to calculate mean nadir GH, which has been demonstrated by Faje et al. [32] to be effectively correlated to IGF-I levels, due to the too low intensiveness.

Moreover, we did not utilize a modern platform for IGF-I measurement incorporating LC/MS: this could possibly impact the applicability of the results.

Eventually, the cross-sectional design of the study did not allow a longitudinal investigation whether patients with ‘high GH’, as SGH or as GHP, actually would show some difference in acromegaly disease history.

In conclusion, the high sensitivity and very low specificity of the SGH value does not seem to make it a reliable test to assess the control of disease during SRLs therapy and eventually to decide a titration in therapy. When an additional parameter to IGF-I, considered the most reliable in the monitoring of therapy, is necessary in defining disease control, the use of BMI-related cut-off and/or multiple determinations (as in GHP) should be taken into consideration.
